# Tumor and Endothelial Cell Hybrids Participate in Glioblastoma Vasculature

**DOI:** 10.1155/2014/827327

**Published:** 2014-04-24

**Authors:** Soufiane El Hallani, Carole Colin, Younas El Houfi, Ahmed Idbaih, Blandine Boisselier, Yannick Marie, Philippe Ravassard, Marianne Labussière, Karima Mokhtari, Jean-Léon Thomas, Jean-Yves Delattre, Anne Eichmann, Marc Sanson

**Affiliations:** ^1^Sorbonne Universités, UPMC, Université Paris 06, Inserm, CNRS, UM 75, U 1127, UMR 7225, ICM, 75013 Paris, France; ^2^UMR911-CRO2, Faculté de Médecine de la Timone, Université de la Méditerranée, 13000 Marseille, France; ^3^AP-HP, Groupe Hospitalier Pitié-Salpêtrière, Service de Neurologie Mazarin, 75013 Paris, France; ^4^AP-HP, Groupe Hospitalier Pitié-Salpêtrière, Service de Neuropathologie Raymond Escourolle, 75013 Paris, France; ^5^INSERM U833, Collège de France, 75005 Paris, France

## Abstract

*Background.* Recently antiangiogenic therapy with bevacizumab has shown a high but transient efficacy in glioblastoma (GBM). Indeed, GBM is one of the most angiogenic human tumors and endothelial proliferation is a hallmark of the disease. We therefore hypothesized that tumor cells may participate in endothelial proliferation of GBM. *Materials and Methods.* We used *EGFR* FISH Probe to detect *EGFR* amplification and anti-CD31, CD105, VE-cadherin, and vWF to identify endothelial cells. Endothelial and GBM cells were grown separately, labeled with GFP and DsRed lentiviruses, and then cocultured with or without contact. *Results.* In a subset of GBM tissues, we found that several tumor endothelial cells carry *EGFR* amplification, characteristic of GBM tumor cells. This observation was reproduced *in vitro*: when tumor stem cells derived from GBM were grown in the presence of human endothelial cells, a fraction of them acquired endothelial markers (CD31, CD105, VE-cadherin, and vWF). By transduction with GFP and DsRed expressing lentiviral vectors, we demonstrate that this phenomenon is due to cell fusion and not transdifferentiation. *Conclusion.* A fraction of GBM stem cells thus has the capacity to fuse with endothelial cells and the resulting hybrids may participate in tumor microvascular proliferation and in treatment resistance.

## 1. Introduction

Glioblastomas (GBM) are the most frequent and malignant primary brain tumors in adults with poor prognosis despite surgery and conventional radio-chemotherapy. Histologically, GBM are highly angiogenic and characterized by microvascular proliferations (previously called endothelial cell proliferations) typically consisting on multilayered tufts of mitotically active endothelial cells together with smooth muscle cells and pericytes [1]. Among targeted therapies tested to date, only antiangiogenic drugs and particularly anti-VEGF have shown efficacy with a nearly 50% of responders [2, 3]. However, this effect is always transient suggesting that GBM can acquire secondary antiangiogenic resistance. Therefore, understanding tumor endothelial cell abnormalities is important to optimize therapy. It is well established that tumor blood vessels differ from normal vessels by altered morphology, blood flow, permeability, and basement membrane deposition [4–7]. Furthermore, evidence indicates that tumor endothelial cells overexpress specific genes, proliferate rapidly, and are sensitive to growth factors and resistant to chemotherapeutic drugs [8–12]. Surprisingly, tumor endothelial cells can harbor the same chromosomal abnormality as tumor cells in B-cell lymphomas [13], multiple myeloma [14], and neuroblastoma [15], suggesting a tumor origin of at least a fraction of intratumoral endothelial cells. Another subpopulation of tumor cells possesses characteristics associated with normal stem cells, specifically the ability to give rise to all cell types found in a particular cancer sample [16]. In brain tumors, including GBM, tumor stem cells have been shown to possess marked capacity for proliferation, self-renewal, and differentiation into all neural lineages [17]. It has also been suggested that normal mouse neural stem cells cocultured with human endothelial cells convert into endothelial cells by transdifferentiation [18].

We here investigated whether endothelial cells of tumor origin might be present in human GBM samples and whether these cells derive from GBM stem cells. Analysis of* EGFR* amplified human GBM tissues by fluorescent* in situ* hybridization (FISH) combined with immunophenotyping [15] showed rare endothelial cells exhibiting* EGFR* amplification. We then investigated the capacity of GBM stem cells (GSC) to acquire an endothelial phenotype* in vitro* and demonstrate that this property results from cell fusion and not transdifferentiation.

## 2. Materials and Methods

### 2.1. GBM Tissue Preparation

Formalin-fixed, paraffin-embedded tissue sections (5 *μ*m) from 10 GBM tumors (World Health Organization classification of brain tumors [1]) carrying* EGFR* amplification identified by CGHa analysis [19] were deparaffinized twice with xylene. The slides were subsequently rehydrated in a series of ethanol solution (100%, 90%, and 70%), washed with phosphate-buffered saline (PBS), and treated with antigen retrieval solution (citrate buffer pH 9.0; Dako Cytomation, France) at 96°C for 20 minutes.

### 2.2. Fluorescent* In Situ *Hybridization and Immunofluorescence

Briefly, slides were blocked with PBS/3% bovine serum albumin (BSA) for 20 minutes and immunostained with mouse anti-human CD31 (1 : 20; Dako Cytomation, France) for 30 minutes at room temperature. Alexa 488-conjugated goat anti-mouse antibody (1 : 1000; Molecular Probes, Invitrogen, France) was added as secondary reagent. After immunostaining, slides were washed three times for 5 minutes each in PBS containing 0.5% Tween-20 (Sigma-Aldrich, France). Only sections of high morphologic quality were used for fluorescent* in situ* hybridization (FISH). The* EGFR* FISH Probe Mix (Dako Cytomation, France) was used according to the manufacturer's instructions. Slides were dehydrated through a series of ethanol washes (70%, 90%, and 100%), denatured in the presence of the specific probes at 82°C for 5 minutes, and incubated overnight in a humid chamber at 45°C. Posthybridization washes were performed, and the slides were mounted in antifade medium Fluoromount-G (Interchim, France) with DAPI (Sigma-Aldrich, France). Slides were analyzed using a Zeiss AxioImager.Z1 microscope.

### 2.3. Cell Cultures

#### 2.3.1. Culture of Primary GBM Stem Cells and Sphere Forming Assay

GBM samples were provided by the local Department of Neurosurgery from informed and consenting patient, as approved by the local Research Ethics Boards at the Salpetriere Hospital. Histologic analyses were done by the Department of Neuropathology. Samples were washed with Hanks' balanced salt solution (Invitrogen, France), dissected, sectioned, and enzymatically dissociated with both 5 mg/mL of Trypsin (Sigma-Aldrich, France) and 200 U/mL of DNAse (Sigma-Aldrich, France) for 10 minutes at 37°C. Erythrocytes were lysed using NH_4_Cl. Cells were then seeded into T75 flasks at 10000 cells/cm^2^. Culture medium (neurosphere medium) consisted of DMEM/F12 (Invitrogen, France) supplemented with 20 ng/mL of epidermal growth factor (EGF), 20 ng/mL of basic fibroblast growth factor (bFGF; both from Sigma-Aldrich, France), B27 (1 : 50; Invitrogen, France), and 1% Penicillin/Streptomycin. Cultures were incubated in 5% CO_2_ at 37°C. After 3 days of culture, CD133 Microbead Kit (Miltenyi Biotech, France) was used to isolate CD133^+^ tumor cell population according to the manufacturer's instructions. Sorted cells were resuspended in neurosphere medium and maintained in 5% CO_2_/95% O_2_ atmosphere at 37°C. Formed primary spheres were harvested, dissociated into single cells, and plated at the density of 5000 cells/cm^2^ in neurosphere medium. Cultures were fed by changing half of the medium every 3 days. Subsphere-forming assay (also called passage) was repeated every 10 days.

#### 2.3.2. Human Endothelial Cell Culture

Purified human umbilical vein endothelial cells (HUVEC) and human umbilical artery endothelial cells (HUAEC) were obtained from Promocell. Immortalized human cerebral microvascular endothelial cells (hCMEC) were obtained from Dr. Pierre Olivier Couraud (Institut Cochin, France). Endothelial cells were cultured with endothelial cell growth medium EGM-2 (Lonza, France) that contains vascular endothelial growth factor (VEGF), basic fibroblast growth factor (bFGF), epidermal growth factor (EGF), insulin growth factor I (IGF-I), hydrocortisone, ascorbic acid, and 2% fetal bovine serum.

### 2.4. Differentiation Assay of Tumor Spheres

Primary GBM spheres were plated onto sterile multiwell glass slide coated with poly-L-ornithine (Sigma-Aldrich, France) in neurosphere medium lacking EGF and bFGF but supplemented with 10% fetal bovine serum. Cells were fixed after 7 days of differentiation culture with 4% paraformaldehyde for 15 minutes, blocked with PBS/3% BSA for 20 minutes, and immunostained for 1 hour with primary antibodies against nestin (1 : 200; mouse monoclonal IgG1; Santa Cruz Biotechnology, Germany), GFAP (1 : 400; rabbit polyclonal; Dako Cytomation, France), and neuronal class III *β*-tubulin (Tuj1; 1 : 500; mouse monoclonal IgG2a; Covance, France). After washes, appropriate secondary antibodies were incubated for 1 hour (1 : 1000; Alexa 594 goat anti-mouse IgG1, Alexa 488 goat anti-rabbit and Alexa 594 goat anti-mouse IgG2a from Molecular Probes, Invitrogen, France). DAPI (Sigma-Aldrich, France) was used for nuclei staining. Slides were mounted in antifade medium Fluoromount-G (Interchim, France) and examined under a Zeiss AxioImager.Z1 microscope.

### 2.5. Endothelial and GBM Cells Cocultures

#### 2.5.1. Noncontact Coculture

Cocultures were prepared on Transwell inserts with 0.4 *μ*m pore size and 6.5 mm diameter (Corning Incorporated, France) placed in 24 well plates. Briefly, GFP-GSC (p3) were seeded at 2 × 10^4^ cells per well in the bottom wells of Transwells, and human endothelial cells were seeded at a ratio of 1 : 1 in the membrane insert wells. Cells were cultured with EGM-2 medium for 5 days. Cell layer fractions were analyzed using Nikon Eclipse TE2000U fluorescence inverted microscope.

#### 2.5.2. Direct Contact Coculture

Dissociated human endothelial cells (5 × 10^5^) and GFP-GSC (5 × 10^5^) were mixed in EGM-2 medium and seeded into T75 flask. After 3 days of culture, cells were dissociated, plated onto sterile multiwell glass slides (1 × 10^4^ on an 8-well slide), and allowed to adhere for 24 hours prior to staining.

### 2.6. Endothelial Markers Immunofluorescence

After 3–5 days, coculture medium was washed away for 10 minutes with PBS before fixation with 4% paraformaldehyde for 15 minutes. Fixed cells were blocked with PBS/3% BSA for 20 minutes and permeabilized for intracytoplasmic antigen with PBS/0.1% Triton X-100. The following primary antibodies were incubated in blocking solution at room temperature for 1 hour: CD31 (1 : 50; mouse monoclonal IgG1; Dako Cytomation, France), CD105 (1 : 5; mouse monoclonal IgG1; Dako Cytomation, France), VE-cadherin (1 : 50; mouse monoclonal IgG1; eBioscience, France), and vWF (1 : 50; mouse monoclonal IgG1; Dako Cytomation, France). Following 3 washes, Alexa 594 goat anti-mouse IgG (1 : 1000; Molecular Probes, Invitrogen, France) was incubated in blocking solution at room temperature for 1 hour as secondary antibody. Nuclei were stained with DAPI (Sigma-Aldrich, France). Slides were mounted in antifade medium Fluoromount-G (Interchim, France) and examined under a Zeiss AxioImager.Z1 microscope. Fluorescence images were captured using AxioCam MRm camera and analyzed with AxioVision Rel. 4.6 software (Carl Zeiss).

### 2.7. Lentiviral Infection

GFP and DsRed lentivirus vector construction and virus production were performed by Dr. Philippe Ravassard (Pierre and Marie Curie University) as previously described [20]. Dissociated GSC were infected with GFP-expressing retroviral vector and HUVEC were infected with DsRed-expressing retroviral vector. Labeled cells were selected using a fluorescence-activated cell sorter (FACS Aria, BD Biosciences). The efficiency of transduction was over 80%.

### 2.8. Cell Sorting

Freshly dissociated cells from GSC-GFP and HUVEC-DsRed cocultures were resuspended in PBS/0.5%BSA/2 mM EDTA solution. Labeled cells were sorted using a fluorescence-activated cell sorter (FACS Aria, BD Biosciences) on the basis of single-cell viability and the presence of double-positive GFP and DsRed fluorescence.

### 2.9. Reverse Transcription-PCR

Total RNA was extracted from GSC and hCMEC using RNable (Eurobio, France) and verified by electrophoresis on Agilent 2100 Bioanalyzer (Agilent Technologies, France). cDNA was synthesized with 200 units of M-MLV Reverse Transcriptase (Invitrogen, France) in 15 *μ*L of 1x first strand buffer (Promega, France), 2 mmol/L deoxynucleotide triphosphates in the presence of 40 units RNase inhibitor RNasin (Promega), 0.5 *μ*g random primers (Promega), and 1 *μ*g total RNA. Semiquantitative PCR amplifications were done with the following primer sequences: CD31 forward 5′-TCCGGATCTATGACTCAGGG-3′ and reverse 5′-ACAGTTGACCCTCACGATCC-3′; VE-cadherin forward 5′-TCCTCTGCATCCTCACTATCACA-3′ and reverse 5′-GTAAGTGACCAACTGCTCGTGAA-3′; ALAS forward 5′-TGCAGTCCTCAGGGCAGTCT-3′ and reverse 5′-TGGCCCCAACTTCCATCAT-3′ as control. The PCR conditions were as follows: 5 minutes at 94°C for denaturation, followed by 30 seconds at 94°C, 1 min at 60°C, and 1 min 30 sec at 72°C for 35 cycles and 7 min at 72°C for final elongation. The RT-PCR products were electrophoretically analyzed in 1% agarose and visualized by ethidium bromide staining.

## 3. Results

### 3.1. Several Intratumoral Endothelial Cells Harbor EGFR Amplification

**Figure 1:**
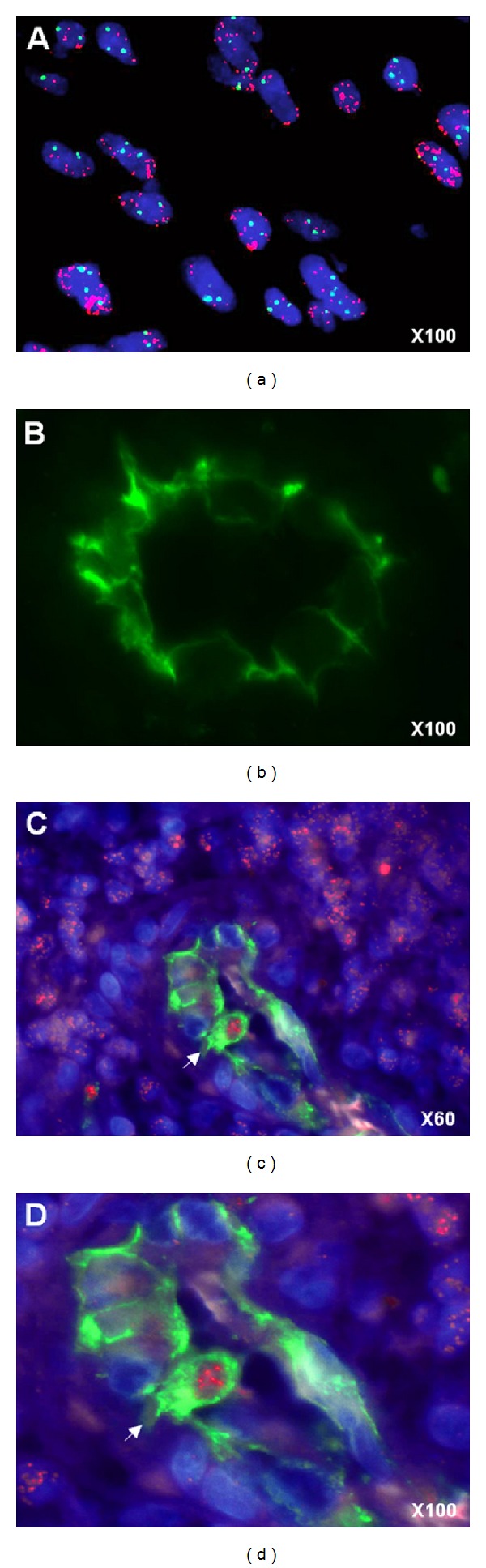
Glioblastoma-derived endothelial cells. (a) Nuclei are stained with DAPI (blue), FISH* EGFR* probe (red) label tumor cells carrying* EGFR* amplification as double minutes (display multiple red signals). (b) Endothelial cells are detected by anti-CD31 immunofluorescence staining. ((c), (d)) A CD31+ (green) endothelial cell (arrow) carrying* EGFR *amplification (multiple red signals) is visible in a GBM microvessel.

We analyzed paraffin sections from 10 GBM with* EGFR* amplification, retrieved from our CGHa database [19], using CD31 monoclonal antibody to identify endothelial cells and FISH detection of* EGFR* amplification to identify tumor cells. As shown in Figure 1(a),* EGFR* amplification was detected as double minutes by red hybridization signals in a large proportion of cells present in GBM tissues. CD31 monoclonal antibody stained endothelial cells but not tumor cells (Figure 1(b)). Only 6 out of 10 GBM sections presented dense microvascular network with high morphologic quality after CD31 immunofluorescence and were then subjected to* EGFR* FISH. Endothelial cells carrying* EGFR* amplification, as shown in Figures 1(c) and 1(d), were identified in 3 out of these 6 GBM tumors. This event was episodic as we were able to count less than a hundred of CD31-*EGFR* FISH costained cells per section. Thus, we identified a minority of tumor endothelial cells that could derive from primary GBM cells.

### 3.2. Glioblastoma Stem Cells Acquire an Endothelial Phenotype When Cocultured with Human Endothelial Cells

**Figure 2:**
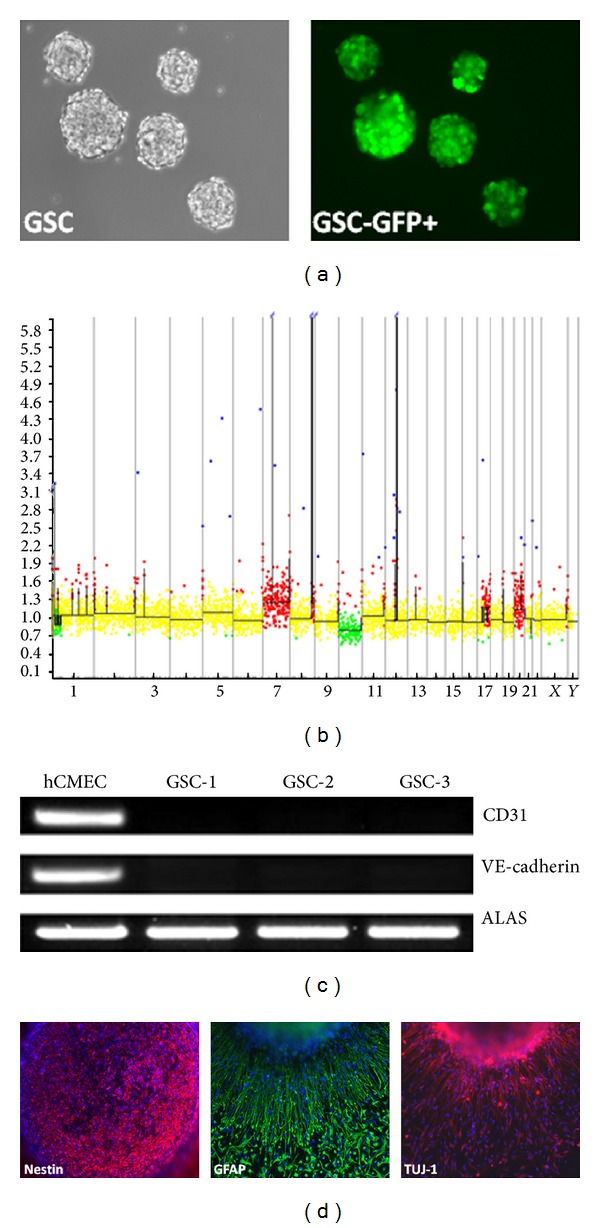
Glioblastoma stem cell characterization. (a) Phase contrast and fluorescent microscopy of GSC-1-GFP+ growing into tumor spheres in neurosphere medium (magnification ×10). (b) Comparative genomic hybridization array (CGHa) of GSC-1 demonstrating tumor genomic alterations. Each BAC (bacterial artificial chromosome) spotted on the comparative genomic hybridization array is represented by a dot. BACs are ordered on the* x*-axis according to their position in the genome. For each chromosome, the telomere of the short arm is on the left and the telomere of the long arm is on the right. The* y*-axis corresponds to fluorescence ratio. Yellow, green, and red indicate, respectively, genomic copy number normal, loss, and gain. Genetic alteration includes complete chromosome 10 loss (green) and gain of chromosome 7 with* EGFR* amplification (arrow). (c) RT-PCR analysis showing specific expression of endothelial markers CD31 and VE-cadherin by hCMEC and not by GSC-1, GSC-2, and GSC-3 after differentiation in EGM-2 medium. ALAS is used as control. (d) Immunostaining of tumor sphere cells for neural stem cell marker (nestin) at the beginning of the differentiation assay, then astrocytic (GFAP) and neuronal (TUJ-1) markers by the differentiated cells around a tumor sphere at day 7 (magnification ×10).

We established 3 GBM stem cell primary cultures (GSC-1, GSC-2, and GSC-3) that demonstrated growth into tumor spheres (Figure 2(a)). They were generated from solid primary adult GBMs carrying* EGFR* amplification and showed conservative genomic profile in culture (Figure 2(b)). Undifferentiated tumor spheres immunostained for nestin (a characteristic neural stem cell marker) and revealed multilineage potential (expression of GFAP for astrocytes and TUJ-1 for neurons) in the differentiation assay as shown in Figure 2(d). We next explored the ability of GSC to transdifferentiate into endothelial cells. None of them expressed or stained with CD31 and VE-cadherin endothelial markers even after growth in EGM-2 medium, known to promote endothelial differentiation [21] (Figure 2(c)).

**Figure 3:**
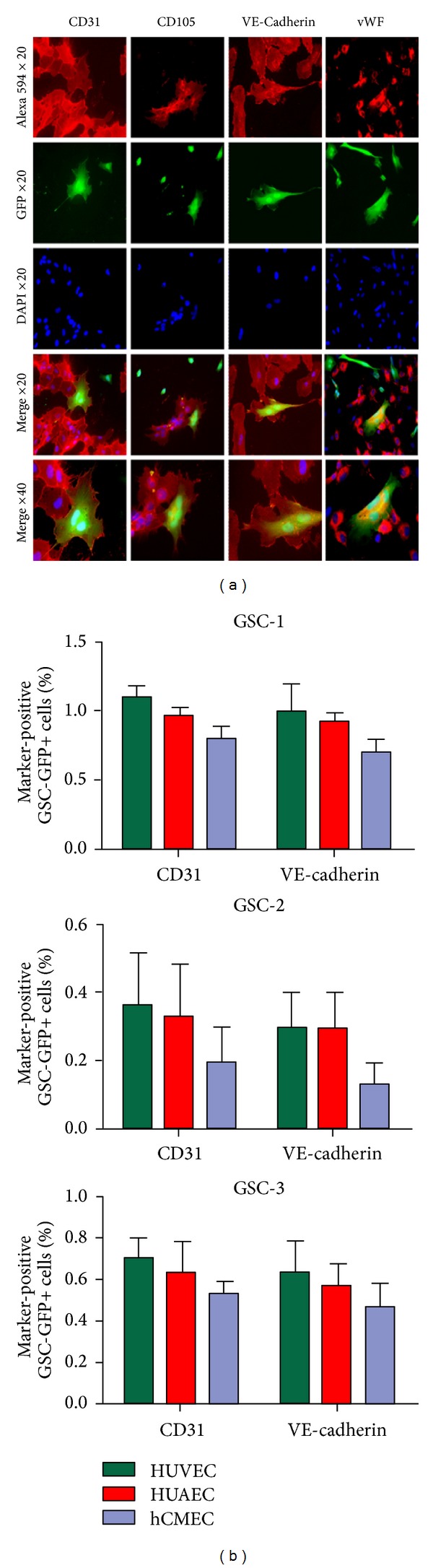
A subset of glioblastoma stem cells acquires an endothelial phenotype. (a) When GSC-GFP were cocultured with human endothelial cells, several multinucleated and cobblestone GSC-GFP expressing CD31, CD105, VE-cadherin, and vWF were observed (b). The percentage of GSC-GFP expressing CD31 and VE-cadherin obtained in coculture with HUVEC, HUAEC, and hCMEC is indicated for GSC-1, GSC-2, and GSC-3.

To test the hypothesis that GSC might need a more specific microenvironment for endothelial conversion, we cocultured GSC-GFP with different human endothelial cells (HUVEC, HUAEC, and hCMEC) allowing cell-cell contact. In this condition, some GSC-GFP exhibited cobblestone morphology and expressed specific endothelial markers including CD31, CD105, VE-cadherin, and vWF (Figure 3(a)). The endothelial-like GSC-GFP were found consistently, but their percentage was variable (0.2% to 1%) depending on the GSC and the endothelial cell types used in direct coculture (Figure 3(b)). However, this conversion was not observed in noncontact coculture, suggesting the implication of cell fusion or cell contact factors.

### 3.3. Some Glioblastoma Stem Cells Acquire the Endothelial Phenotype through Cell Fusion

**Figure 4:**
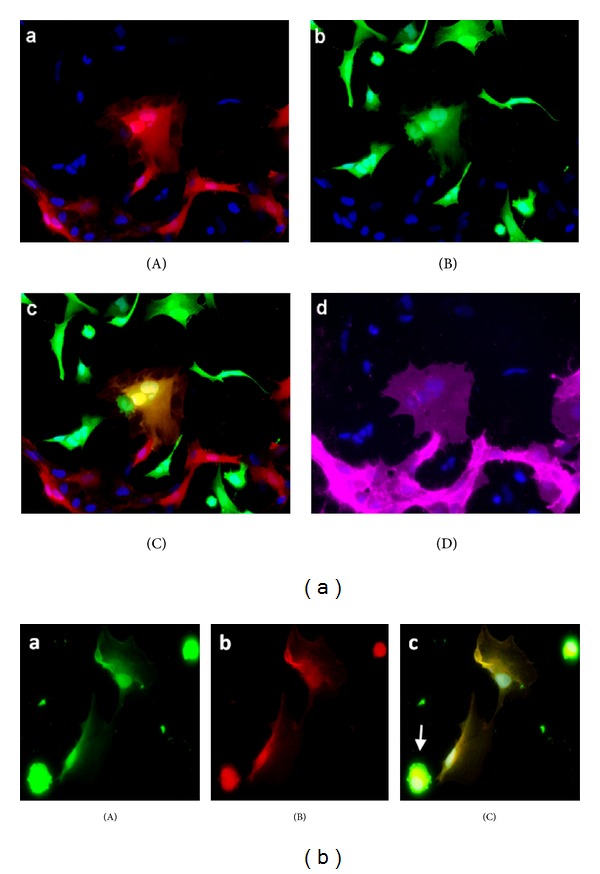
Glioblastoma stem cell fusion with endothelial cells. (a) When GSC-GFP were cocultured with HUVEC-DsRed, we observed binucleated cell expressing both DsRed (A) and GFP (B) (merged in (C)) corresponding to hybrid cell and exhibiting endothelial phenotype as shown by CD31 immunostaining (D). (b) After hybrid cell selection by cell sorting, mononuclear hybrids expressing parental GFP (A) and DsRed (B) were observed (merged in (C)). Apoptotic body of died fused cell (arrow).

The fact that most endothelial-like tumor cells were multinucleated suggested that GSC may acquire endothelial phenotype through cell fusion, rather than cell transdifferentiation. To test this hypothesis, we used DsRed protein-expressing HUVEC to detect cell fusion in fluorescent microscopy. When GSC-GFP were cocultured with HUVEC-DsRed, binucleated cells coexpressing both GFP and DsRed were observed (Figure 4(a), (A)–(C)). GFP+/DsRed+ fused cells maintained CD31 expression (Figure 4(a), (D)), meaning that the endothelial phenotype was dominant. We isolated the GFP+/DsRed+ cells by cell sorting and cultured them in EGM-2 medium to monitor their behavior over time. Most of these cells with heterocaryons were quiescent and died after 5 to 7 days of culture. A small fraction (less than 1%) gave rise to viable mononuclear hybrids expressing the parental GFP and DsRed, as shown in Figure 4(b).

## 4. Discussion

As early as 1948, several observations suggested that cancer cells are located in the walls of tumor blood vessels and participate in their assembly [22, 23]. This is known as “mosaic vessels” where tumor cells form a part of the vessel surface, while the remaining part is covered by endothelium [24]. But in this case, tumor cells in apparent contact with the lumen do not show an endothelial phenotype. We report here that human GBM contain some tumor endothelial cells carrying the same cytogenetic abnormality as tumor cells. This phenomenon was reported previously in B-cell lymphomas [13], multiple myeloma [14], and neuroblastoma [15]. Since a common precursor can give rise to hematopoietic lineage and endothelial cells in hematopoietic tumors, a common progenitor targeted by neoplastic transformation and sharing specific genetic abnormalities can differentiate into tumor cells or endothelial cells [13, 14]. This hypothesis is not applicable to solid tumors of nonhematopoietic origin. Alternative explanations include tumor microenvironment [25, 26], tumor cell transdifferentiation, and cell fusion [27]. Our data strongly suggest that GBM stem cell acquires endothelial phenotype through cell fusion and participates in microvascular structures.

This is consistent with a recent study in which human glioma lines marked with DsRed protein were grafted in eGFP-expressing NOD/Scid mouse to study tumor-host interactions [28]. Using fluorescence-activated cell sorting (FACS), this model is allowed to completely separate host cells from tumor cells and to detect double-positive cells, possibly arising from cell fusion events. Furthermore, CD31 expression was found in 0.3% of separated tumor cells when phenotyped [28]. In normal brain, neural stem cells exist in vascular niches where endothelial cells provide direct cell contacts and secreted factors that regulate neural stem cell function [29, 30]. Similarly, GBM stem cells are maintained within vascular niches that mimic the neural stem cell niche [31]. Nestin+/CD133+ cells within sections of human GBM are located in close proximity to tumor capillaries [31]. This organization can favor cell fusion by facilitating cell contact between GBM stem cells and endothelial cells.

Cell fusion has been debated in adult stem cells plasticity. Several reports revealed the ability for stem cells to change fate through cell fusion rather than cell transdifferentiation. New hybrids resulting from nuclear fusion can generate mononuclear differentiated progeny that exhibit both parental phenotypes [18, 32–35]. These findings raise the possibility that cell fusion has undiscovered functions which can promote disease, especially cancer. The idea that cell fusion contributes to cancer progression was introduced almost 100 years ago with a proposal that malignancy is a consequence of hybridization between leukocytes and somatic cells [36]. Sixty years later, this idea was expanded by proposing that hybridization of tumor cells with lymphocytes results in metastatic cells [37] and that cell fusion promotes the phenotypic and genotypic diversity of tumors [38]. The best defended theory is cancer cell fusion with macrophages or other migratory bone marrow-derived cells which provides a unifying explanation for metastasis [39]. Although host cell-cancer cell fusion has been demonstrated and well documented in animals [40], there is as yet far less information on human cancer. A few human cases have recently been reported [41–45], but it is unclear whether the scarcity of reports on cell fusion in human tumors is due to rarity of this event or to a difficulty to detect it.

In addition to cell fusion, we cannot exclude that transdifferentiation of GBM stem cells into endothelial cells may exist. Conversion from GBM to endothelial phenotype was not observed in noncontact coculture, suggesting either cell fusion or cell-cell contact mediated transdifferentiation. Recently two recent papers showed that transdifferentiation of GBM stem cells into endothelial cells does exist in GBM (p7). Notch pathway is involved in this mechanism [46–48].

Considering that cell fusion can be linked to several fundamental features of cancer, more investigations are needed to clarify this emergent concept of “hidden enemy” in cancer pathology. In addition, the presence of these hybrid cells in GBM endothelium supports new therapeutic approaches, such as intravascular targeted strategy directed against the EGFRVIII mutant, a specific antigen expressed by 20–30% of GBM [49].
